
JOURNAL INFORMATION
==============================
NLM Title Abbreviation: ScientificWorldJournal
Journal ID: ScientificWorldJournal
NLM Journal ID: 101131163
Journal ID: TSWJ

The Scientific World Journal

ISSN: 2356-6140
EISSN: 1537-744X
Publisher: Wiley

ARTICLE INFORMATION
==============================
PMCID: PMC6084217
PMID: 30147533
DOI: 10.1100/tsw.2001.166
Article ID: 829683
Article version: 1
Subjects: Short Report

Actinomycin D Induces Hepatocyte Cell Death and Upregulation of FADD/MORT1

Kim Peter K.M. *
Billiar Timothy R.
Department of Surgery, University of Pittsburgh Medical Center, W1503 Biomedical Science Tower, 200 Lothrop St., Pittsburgh, PA 15261, USA
*Peter K.M. Kim: kimp@msx.upmc.edu
Publication date: 2001
Electronic publication date: 2001 Jun 8
Volume: 1
First page: 61
Last page: 61
Copyright: Copyright © 2001 Peter K.M. Kim and Timothy R. Billiar.
Copyright year: 2001
License: This is an open access article distributed under the Creative Commons Attribution License, which permits unrestricted use, distribution, and reproduction in any medium, provided the original work is properly cited.
License URL: https://creativecommons.org/licenses/by/3.0/

Funding: National Institutes of Health R01-GM-44100

==============================
Miami *Nature Biotechnology* Short Reports
*TheScientificWorld* (2001) 1(S3), 61SR
ISSN 1532-2246; DOI 10.1100/tsw.2001.166

### ACTINOMYCIN D INDUCES HEPATOCYTE CELL DEATH AND UPREGULATION OF FADD/MORT1

Peter K.M. Kim* and Timothy R. Billiar

Department of Surgery, University of Pittsburgh Medical Center, W1503 Biomedical Science Tower, 200 Lothrop St., Pittsburgh, PA 15261
* KIMP@MSX.UPMC.EDU

**INTRODUCTION.** We have studied ligand-dependent apoptosis via the tumor necrosis factor (TNF) receptor in hepatocytes. (1) These experiments require the addition of actinomycin D (ActD) to kill cells because TNF alone does not kill rat hepatocytes in culture. FADD/MORT1 (fas-associated death domain protein) is an intracellular protein that mediates activation of proteases called caspases which are critical to the propagation of the apoptotic signaling cascade. (2) Because of its pivotal role in the cell death pathway of multiple receptors (3), we believe that the regulation of FADD may have profound effects on the modulation of cell death. We have studied the effects of actinomycin D on FADD in primary rat hepatocyte cultures in order to elucidate the molecular pathways of cell death that may be altered or manipulated in cancer.

**METHOD.** Hepatocytes from Sprague-Dawley rats were isolated by perfusion of livers with collagenase and cells were plated on a collagen matrix at $10^6$ cells per ml in Williams E media containing 10% calf serum. Cells were treated with fresh media containing ActD (200 ng/ml), TNF (2000 units/ml), and/or caspase inhibitors (Z-VAD 100 μM, Z-DEVD-fmk 100 μM), or Z-IETD-fmk 100 μM). Twelve hours later, cells were harvested by scraping. Protein lysates were obtained by freezing and thawing the cells in hypotonic buffer containing protease inhibitors. Viability was determined using crystal violet staining of cells that remained adherent to the plates, lysis with 1%SDS, then quantitation by spectrophotometry at 550 nm. Western blots were performed by running protein on 13%SDS-PAGE gels, transferring to nitrocellulose membranes, then probing with anti-murine FADD antibody.

**RESULTS.** Western blotting showed that FADD protein was increased in primary rat hepatocyte cultures by actinomycin D, but not by TNF alone. (Figure 1a) Viability as measured by crystal violet staining of rat hepatocytes inversely correlated with the increase in FADD seen with 12 hour stimulation with 200 ng/ml actinomycin D - 44% of the cells survived compared to media stimulation alone. (Figure 1b) This response is dose-dependent and time-dependent; upregulation of FADD protein can be seen with as little as 12.5 ng/ml actinomycin D for 8 hours or 200 ng/ml for 4 hours. The increase in FADD protein with actinomycin D is similar to the effect seen with actinomycin D and TNF together. (Figure 1a) The pan-caspase inhibitor Z-VAD, caspase 3 inhibitor DEVD, and caspase 8-inhibitor Z-IETD prevented cell death by actinomycin D, but did not prevent the increase in FADD protein as seen by western blotting (Figure 2a, 2b).

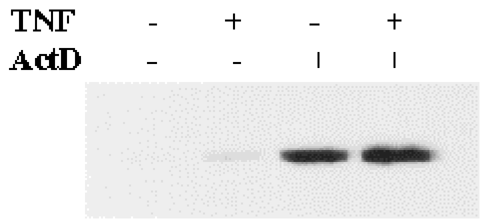

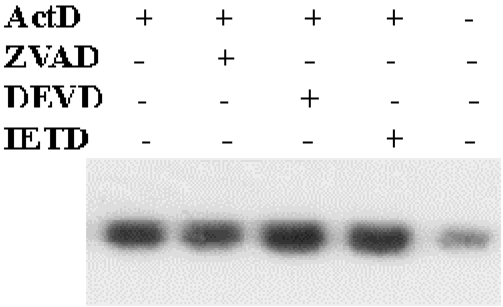

**DISCUSSION.** The killing of rat hepatocytes by actinomycin D is associated with an increase in FADD protein in a dose- and time-dependent manner. Caspase inhibitors prevented cell death by actinomycin D without blocking the increase in FADD expression. Actinomycin D, which inhibits transcription by intercalating DNA, is a commonly used chemotherapeutic agent for Wilm's tumor, rhabdomyosarcoma, and choriocarcinoma. These results suggest that the increase in expression of FADD by actinomycin D initiates the cell death cascade by a mechanism upstream of any caspase activation that does not require Fas or TNF. The upregulation of FADD may contribute to the effects of actinomycin D as a chemotherapeutic drug.

**ACKNOWLEDGEMENTS.** This work was supported by National Institutes of Health Grant R01-GM-44100 (T.R.B.).

**REFERENCES.**
1. Li, J., Bombeck, C.A., Yang, S., Kim, Y.M., and Billiar, T.R. (1999) J. Biol. Chem. 274, 17325-17333
2. Kischkel, F.C., Hellbardt, S., Behrmann, I., Germer, M., Pawlita, M., Krammer, P.H., and Peter, M.E. (1995) EMBO J. 14, 5579-5588
3. Ashkenazi, A. and Dixit, V.M. (1998) Science 281, 1305-1309
